# The cardiometabolic index as a predictor of sleep disorders and mortality: A cross-sectional study

**DOI:** 10.1097/MD.0000000000042029

**Published:** 2025-04-04

**Authors:** Zhisen Xu, Xuanfang Qian, Junyao Xu

**Affiliations:** a Harbin 242 Hospital, Harbin, China; b Zhejiang Cancer Hospital, Hangzhou Institute of Medicine (HIM), Chinese Academy of Sciences, Hangzhou, Zhejiang, China; c Zhejiang Chinese Medical University, Hangzhou, China.

**Keywords:** CMI, mortality, national survey, sleep disorders

## Abstract

The cardiometabolic index (CMI) reflects an individual’s cardiometabolic health and is linked to the risk of dyslipidemia, obesity, hyperglycemia, and hypertension. These risk factors not only increase the likelihood of cardiovascular disease but are also strongly associated with sleep issues such as sleep apnea and insomnia. However, the relationship between CMI and the risk of sleep disorders remains unclear. This study aimed to investigate the association between CMI and sleep disorder-related morbidity and mortality. This cross-sectional study utilized data from 6220 adults aged ≥ 20 years from the National Health and Nutrition Examination Survey (2007–2014). The CMI was calculated as [waist circumference (cm)/height (cm)] × [triglycerides (mmol/L)/high-density lipoprotein-C (mmol/L)], reflecting metabolic risk. Participants were categorized into 3 CMI tertiles (Q1–Q3). Based on survey data, participants were classified into sleep disorder and non-sleep disorder groups. The analysis included logistic regression, subgroup analysis, forest plots, and survival analysis. The average age of participants was 49 ± 18.00 years; 49% were male. The high-CMI group had older participants, more males, higher body mass index, higher triglycerides, and more hypertension (*P* < .001). Higher CMI was significantly associated with an increased risk of sleep disorders (odds ratio [OR] = 1.11, 95% CI: 1.02 to 1.21, *P* = .017), with the prevalence being greater in Q3 than in Q1 (OR = 1.46, 95% CI: 1.27 to 1.68, *P* ≤ .001). After adjusting for demographics, the association persisted (OR = 1.13, 95% CI: 1.03–1.24, *P* = .014). The mortality rate was also higher in the high-CMI group (*P*≤.001), with a 34% increased risk of death (OR = 1.34, 95% CI: 1.08–1.67, *P* = .021). The study found that a higher CMI is associated with increased risks of sleep disorders and mortality. Understanding this relationship may help in monitoring cardiometabolic health and assessing sleep disorder severity. CMI could serve as a cost-effective indicator for sleep disorder assessment.

## 1. Introduction

Sleep disorders are important public health problems that are gradually being taken increasingly seriously.^[1]^ Recent data from the Centers for Disease Control and Prevention based on the 2014 Behavioral Risk Factor Surveillance System revealed that the median prevalence of adults sleeping <7 hours was 35.1% across the United States, ranging from 28.4% to 43.9% in different states. Additionally, the prevalence of extremely short sleep duration (5 hours or less) was 11.8%, highlighting the significant burden of insufficient sleep among American adults.^[2]^ Not only do sleep disorders reduce the quality of life, but they also have significant implications for overall health, contributing to the development of chronic conditions such as cardiovascular disease, diabetes, and depression.^[3–5]^ Recent evidence suggests that sleep disorders and cardiometabolic diseases often coexist and share common risk factors, including unhealthy dietary patterns, psychological stress, obesity, and physical inactivity.^[6–8]^ These shared factors exacerbate metabolic dysregulation and disrupt sleep homeostasis, creating a bidirectional relationship between the 2 conditions. Understanding the factors that contribute to sleep disorders is critical for identifying effective interventions and promoting public health. Addressing these shared risk factors may provide new insights into mitigating the burden of both cardiometabolic diseases and sleep disorders.

The cardiometabolic index (CMI) is considered a new method for estimating visceral adipose tissue and was originally introduced by Ichiro Wakabayashi in 2015.^[9]^ Because it combines indices of abdominal obesity and dyslipidaemia, the CMI has been shown to be a powerful and independent discriminator of clinical obesity. The CMI can not only reflect cardiometabolic risk factors such as dyslipidaemia, obesity, hyperglycemia, and hypertension but also reflect an individual’s cardiometabolic health status.^[10,11]^ Some studies have shown a correlation between this indicator and the risk of developing diseases such as type 2 diabetes, stroke, atherosclerosis of peripheral arterial disease, and depressive mood.^[12–14]^

Some studies have confirmed that cardiometabolic risk factors such as obesity, hypertension and hyperlipidemia not only increase the risk of cardiovascular disease but are also strongly associated with sleep problems such as sleep apnea and insomnia.^[15–17]^ Sleep disorders can also affect metabolic health through a variety of mechanisms, including disrupted hormone production, insulin resistance, and an increased inflammatory response.^[18–20]^ However, no relevant studies have been conducted to specifically examine the relationship between the CMI and the risk of developing sleep disorders. The aim of the present cross-sectional study was to fill that gap by exploring the relationship between cardiometabolic risk and the risk of developing sleep disorders. In this study, we provide new insights into cardiometabolic health monitoring and sleep disorder severity. The CMI might be a convenient and cost-effective indicator for assessing this disease.

## 2. Methods

### 2.1. Research population

In this cross-sectional study, we utilized data from the National Health and Nutrition Examination Survey (NHANES). The NHANES program is designed to assess the health and nutritional status of adults and children in the United States (NHANES website: http://www.cdc.gov/nchs/nhanes.htm). The data used in this study were selected from 4 NHANES cycles from 2007 to 2014. In this study, we focused on adult participants aged 20 years and older for whom complete and reliable information (demographics, body measurements, disease information, etc) was available.

**Figure 1.**
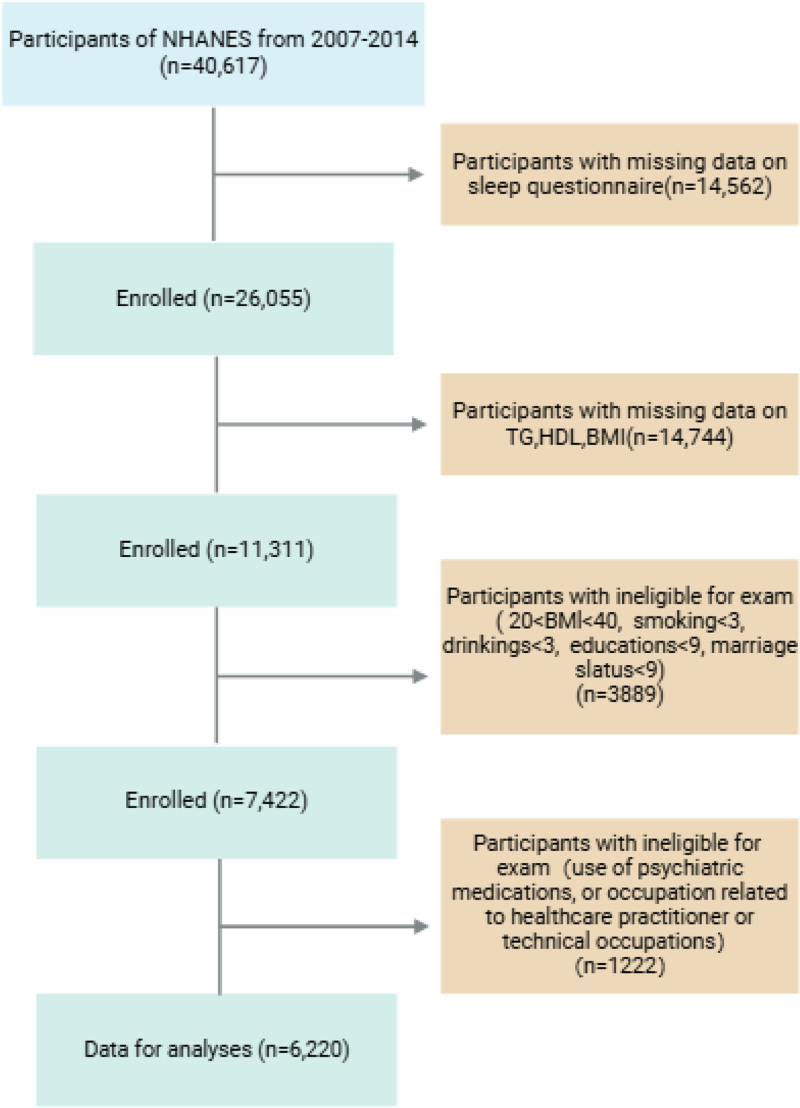
Flowchart of the participant selection from NHANES 2007 to 2014. NHANES = National Health and Nutrition Examination Survey.

The exclusion criteria were (i) missing data from sleep questionnaires, (ii) missing or incomplete data on triglycerides (TG) or high-density lipoprotein (HDL) concentrations or body mass index (BMI), and (iii) missing data on BMI > 40 or BMI < 20 status, smoking status, alcohol use status, marital status, hypertension status, (iv) individuals using psychiatric medications, and healthcare workers who have night shift work, all of whom were excluded from the analysis. Ultimately, we included 6220 participants in our final analysis (Fig. 1).

### 2.2. Sleep disorders

Sleep disorders were the dependent variable for this study. We comprehensively assessed via 3 questions: “How much sleep do you usually get at night on weekdays or workdays?,” “Have you ever told a doctor or other health professional that you have trouble sleeping?,” and “Have you ever been told by a doctor or other health professional that you have a sleep disorder?.” Sleep duration was categorized as short (<7 hours per night), normal (7–9 hours per night), or long (>9 hours per night). The question used to assess sleep difficulties was answered. Sleep factors (sleep duration, self-reported sleep difficulties, and diagnosed sleep disorders) were included in the calculation of the overall sleep score. Low-risk sleep is defined as sleeping 7 to 9 hours per night without sleep difficulties or sleep disorders. Finally, we obtained a total score from 0 to 3, redefined the overall sleep pattern as a “poor sleep pattern” (0 ≤ overall sleep score ≤ 1) or a “moderate, healthy sleep pattern” (overall sleep score = 2, 3), and reclassified the participants into a sleep disorder group or a non-sleep disorder group.

### 2.3. The cardiometabolic index

The cardiometabolic index was the independent variable in this study and was calculated as [waist circumference (cm)/height (cm)] × [TG (mmol/L)/HDL-C (mmol/L)].^[21]^ In the CMI, measures of obesity and lipid metabolism are combined to reflect metabolic risk.

We included covariates in our analyses to account for the confounding effects of other factors. Based on NHANES interviews and examinations and laboratory and questionnaire data, we included the following covariates: age, sex, race, education level, the poverty-to-income ratio (PIR), marital status, BMI, smoking status, drinking status, hypertension status, diabetes mellitus status, triglyceride concentrations, and HDL concentrations. Continuous variables are expressed as the weighted mean ± standard deviation (SD), whereas categorical variables are expressed as weighted percentages (95% confidence intervals, 95% CIs) and were compared using one-way analysis of variance and chi-square tests.

### 2.4. Data analysis

Descriptive statistics were calculated to characterize the participants based on the CMI tertiles. Model 1 was a crude model (unadjusted). Model 2 was adjusted for demographic variables: age, sex, race, education level, the PIR, and marital status. Model 3 was adjusted for age, sex, race, education level, the PIR, BMI, marital status, smoking status, drinking status, hypertension status, and TG and HDL concentrations. Additionally, subgroup analyses were conducted within the fully adjusted model to explore the relationship between sleep status and CMI metric outcomes in the adjusted model.

Because this is a complex sampling design for a national population, we considered 2-year MEC weights, primary sampling units, and sampling stratum in all analyses.^[22]^ In this study, the new 4-year weights can be calculated by dividing the 2-year weights by 2.

Continuous variables were expressed as weighted means ± SD, and categorical variables were expressed as weighted percentages (95% confidence intervals, 95% CIs). Group comparisons were performed using one-way analysis of variance for continuous variables and chi-square tests for categorical variables. Statistical significance was set at *P* < .05. All statistical analyses were conducted using R software (version 4.3.0; R Foundation for Statistical Computing, Vienna, Austria; https://www.R-project.org).

## 3. Results

### 3.1. The baseline characteristics of the study population

This study included 6220 adults with a mean age of 49 ± 18 years, of whom 49% were males and 51% were females. Compared to those with a low CMI, those with a high CMI were older, more likely to be male, had a higher BMI, had higher triglyceride concentrations, and a higher prevalence of hypertension. The detailed results are shown in Table 1.

**Table 1:** CMI characteristics of National Health and Nutrition Examination Survey participants, 2007 to 2014.

Characteristic	N*	Overall	Q1	Q2	Q3	*P*-value‡
		N = 75,866,740†	N = 25,070,607†	N = 25,031,517†	N = 25,764,616†	
*Age*	6220	49 ± (18)	46 ± (18)	49 ± (18)	52 ± (17)	<.001
*Gender*	6220					<.001
Male		3083 (49%)	783 (39%)	1037 (50%)	1263 (59%)	
Female		3137 (51%)	1197 (61%)	1038 (50%)	902 (41%)	
*Race*	6220					<.001
Mexican American		957 (8.5%)	184 (5.5%)	342 (9.0%)	431 (11%)	
Other Hispanic		665 (5.7%)	190 (5.4%)	217 (5.7%)	258 (6.0%)	
Non-Hispanic White		2884 (70%)	869 (68%)	933 (69%)	1082 (72%)	
Other Hispanic		1140 (9.6%)	546 (15%)	379 (9.3%)	215 (4.9%)	
Other race		574 (6.4%)	191 (6.5%)	204 (6.7%)	179 (6.0%)	
*Education*	6220					<.001
≤High school		3008 (40%)	772 (33%)	1040 (41%)	1196 (45%)	
>High school		3212 (60%)	1208 (67%)	1035 (59%)	969 (55%)	
*Marital status*	6220					.008
Non-single		3837 (66%)	1136 (63%)	1293 (65%)	1408 (68%)	
Single		2383 (34%)	844 (37%)	782 (35%)	757 (32%)	
*BMI*	6220	28.2 ± (4.7)	25.6 ± (4.0)	28.3 ± (4.4)	30.6 ± (4.4)	<.001
*Family income*	6220	2.98 ± (1.62)	3.11 ± (1.62)	2.94 ± (1.62)	2.88 ± (1.62)	.003
*Smoking*	6220					<.001
Yes		2568 (40%)	652 (33%)	860 (40%)	1056 (47%)	
No		3652 (60%)	1328 (67%)	1215 (60%)	1109 (53%)	
*Drinking*	6220					.8
Yes		4419 (76%)	1423 (77%)	1462 (76%)	1534 (76%)	
No		1801 (24%)	557 (23%)	613 (24%)	631 (24%)	
*Hypertension*	6220					<.001
Yes		2355 (34%)	561 (23%)	786 (35%)	1008 (44%)	
No		3865 (66%)	1419 (77%)	1289 (65%)	1157 (56%)	
*CMI*	6220	0.75 ± (1.05)	0.24 ± (0.08)	0.52 ± (0.10)	1.48 ± (1.55)	<.001
*HDL-cholesterol* (mmol/L)	6220	1.38 ± (0.40)	1.71 ± (0.39)	1.36 ± (0.27)	1.09 ± (0.23)	<.001
*Triglyceride* (mmol/L)	6220	1.45 ± (1.20)	0.75 ± (0.22)	1.21 ± (0.29)	2.36 ± (1.66)	<.001

BMI = body mass index.

* N not missing (unweighted).

† Mean ± (standard deviation); n (unweighted) (%).

‡ Wilcoxon rank-sum test for complex survey samples; chi-squared test with Rao & Scott second-order correction.

### 3.2. Associations between the CMI and the prevalence of sleep disorders

In the multivariate logistic regressions, the CMI was equalized into 3 quartiles, correcting for the demographic variables of age, sex, ethnicity, educational attainment, household income, and marital status in Model 1. Model 2 was adjusted for age, sex, ethnicity, educational attainment, PIR, marital status, smoking status, drinking status, and HDL-C and triglyceride concentrations.

We found a positive association between the CMI and the prevalence of sleep disorders in the unadjusted model (odds ratio [OR] = 1.11, 95% CI: 1.02–1.21, *P* = .017). The prevalence of sleep disorders was greater in the Q3 subgroup than in the Q1 subgroup (OR = 1.46, 95% CI: 1.27–1.68, *P* < .001). The CMI and the prevalence of sleep disorders were positively correlated in the model adjusted for demographic variables (OR = 1.13, 95% CI: 1.03–1.24, *P* = .014). The prevalence of sleep disorders was greater in the Q3 subgroup than in the Q1 subgroup (OR = 1.56, 95% CI: 1.35–1.81, *P* < .001). No significant correlation was found in the fully adjusted model. The results are shown in Table 2.

**Table 2:** Weighted logistic regression coefficients (OR) and 95% confidence intervals between sleep disorders and CMI: United States, 2007 to 2014.

	Crude model	Model 1	Model 2
	OR (95% CI) *P*-value	OR (95% CI) *P*-value	OR (95% CI) *P*-value
CMI	1.11 (1.02,1.21) .017	1.13 (1.03,1.24) .014	1.04 (0.90,1.20) .6
*CMI tertile*			
Q1	Ref.	Ref.	Ref.
Q2	1.22 (1.04,1.44) .016	1.26 (1.08,1.49) .005	1.13 (0.94,1.35) .2
Q3	1.46 (1.27,1.68) < .001	1.56 (1.35,1.81) < .001	1.26 (0.99,1.61) .056
*P* for trend	<.001	<.001	.056

CMI = cardiometabolic index.

### 3.3. Subgroup analysis

**Figure 2.**
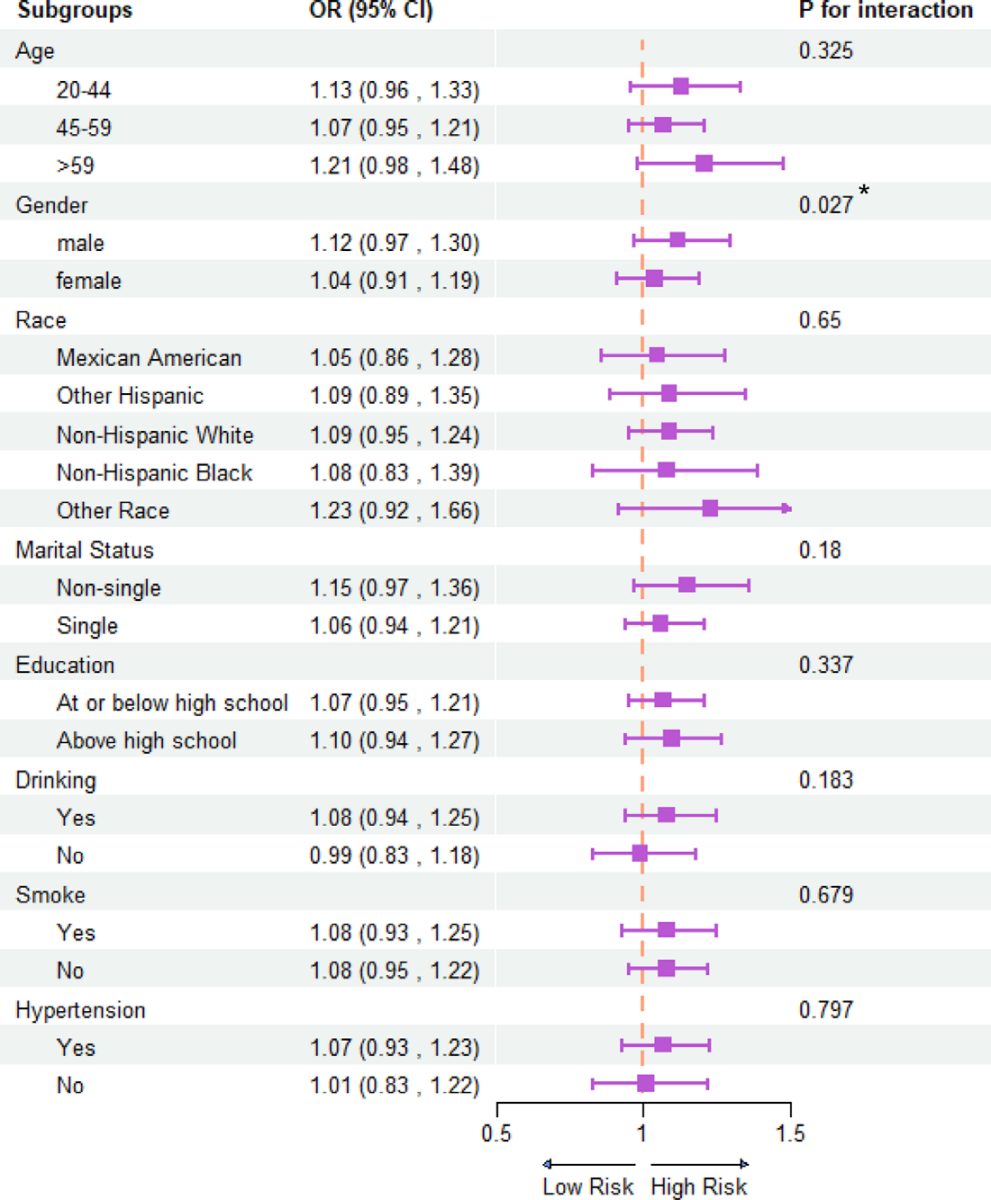
Subgroup analysis for the association between CMI and sleep disorders. Weighted univariate logistic regression was used for subgroup analysis. CMI = cardiometabolic index.

**Figure 3.**
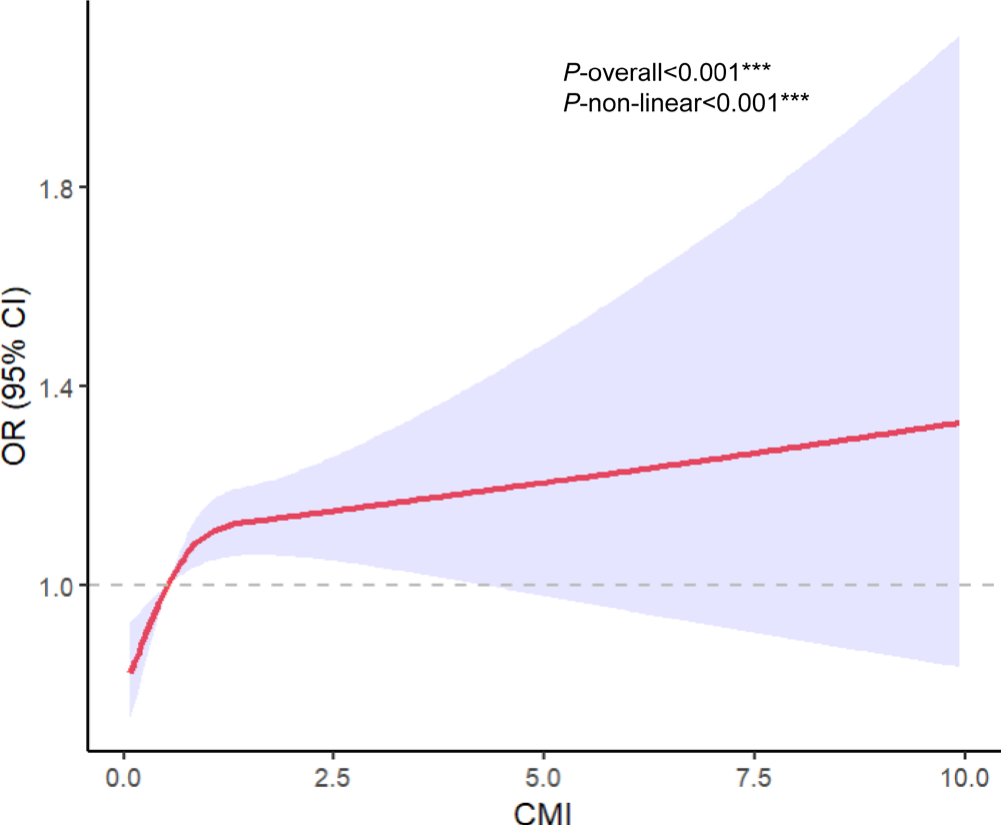
Nonlinear associations between CMI and sleep disorders. Restricted cubic spline for the association between CMI and sleep disorders. CMI = cardiometabolic index, OR = odd ratio.

We performed a subgroup analysis of the fully adjusted model (Fig. 2). The analyses included age (20–44, 45–59, and ≥60 years), sex, race, hypertension status, smoking status, marital status, alcohol use status, and education. Sex was found to influence the association between the CMI and the prevalence of sleep disorders (interaction *P* = .027) (Fig. 2). No significant difference was found for *P* for the interaction of other covariates. Furthermore, the results of the smoothed curve-fitting analysis revealed a positive relationship between the CMI and the prevalence of sleep disorders (Fig. 3).

### 3.4. Correlation between sleep disorders and survival

**Figure 4.**
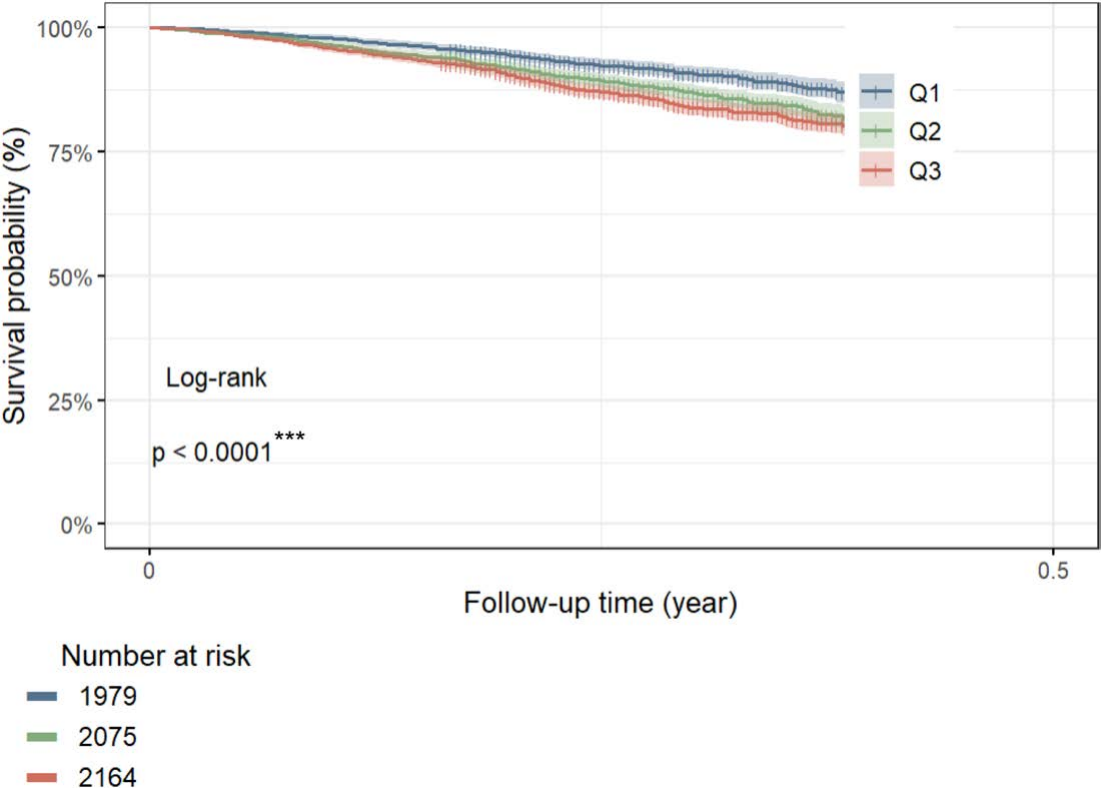
Kaplan–Meier curves of the survival rate with higher (1979), middle (2075) and lower (2164) CMI values. The y-axis indicates the survival rate, and the x-axis indicates the follow-up time (in years). CMI = cardiometabolic index.

We analyzed data for 6220 participants (1979 with a high CMI, 2075 with a moderate CMI, and 2164 with a low CMI) to calculate the association between the CMI and mortality. We observed a positive correlation between the CMI and mortality, with patients in the group with a higher CMI having a greater mortality rate than patients in the group with a lower CMI (*P* < .001) (Fig. 4). Weighted multivariate Cox regression analysis also confirmed the correlation between the CMI and mortality (Table 3). According to the unadjusted model (crude model), each 1-unit increase in the CMI was associated with a 5% increase in the risk of death among respondents (OR 1.05, 95% CI 1.01–1.10; *P* = .004). The group with a high CMI had a 95% greater risk of death than did the group with a low CMI (OR 1.95, 95% CI 1.40–2.38; *P* < .001). After adjusting for confounders (Model 1), the group with a high CMI had a 34% greater risk of death than did the group with a low CMI (OR 1.34, 95% CI 1.08–1.67; *P* = .021) (Table 3).

**Table 3:** Weighted logistic regression coefficients (OR) and 95% confidence intervals between risk of death and CMI: United States, 2007 to 2014.

	Crude model	Model 1	Model 2
	OR (95% CI) *P*-value	OR (95% CI) *P*-value	OR (95% CI) *P*-value
CMI	1.05 (1.02,1.09) .004	1.05 (1.01,1.10) .015	1.11 (0.94,1.32) .224
*CMI tertile*			
Q1	Ref.	Ref.	Ref.
Q2	1.48 (1.17,1.87)	1.10 (0.89,1.36)	1.05 (0.82,1.34)
Q3	1.95 (1.60,2.38)	1.34 (1.08,1.67)	1.27 (0.85,1.90)
*P*-value	<.001	.021	.437

CMI = cardiometabolic index.

## 4. Discussion

In this study, we explored the relationship between the cardiometabolic index (CMI) and the risk of developing sleep disorders using the NHANES database. We found that sleep disorders occurred more commonly in people with a high CMI. After controlling for demographic variables such as age, sex, race, and socioeconomic status, we found that a higher CMI was significantly associated with a greater risk of developing sleep disorders. The adjusted OR showed (Model 2) that for each SD increase in the CMI (Q3 group), there was a 56% increase in the risk of developing sleep disorders. Further interaction analyses revealed a significant interaction effect between the CMI and the risk of developing sleep disorders on a number of key demographic variables, with CMI having larger effect sizes in men than in women. Our study also revealed that the association between the CMI and the risk of developing sleep disorders remained significant after adjusting for possible confounders. This suggests that cardiometabolic health may play an independently important role in sleep disorders. Even after accounting for factors such as socioeconomic status and marital status, the CMI remained a significant predictor of sleep disturbance. Finally, we performed survival analyses and observed a positive correlation between a high or low CM index and mortality, and we found that for every 1-unit increase in the CMI, the risk of death for respondents increased by 5%.

Introduced in 2015, the CMI is a novel marker derived from obesity and lipid profiles. Initially used in diabetes diagnosis,^[9]^ the CMI reflects cardiometabolic risk factors, such as obesity, dyslipidaemia, and hypertension, that may directly affect sleep quality.^[12]^ The CMI has been used in the diagnosis of diabetes mellitus, and high CMI values have been suggested to be a contributing factor to the development of type 2 diabetes mellitus, with a significant correlation between the CMI and hyperuricemia status, as well as studies confirming that CMI can also be used as a predictive marker for the onset of NAFLD.^[23]^ There is still a paucity of literature on sleep and CMI, so our findings help address this gap.

Previous studies have shown that within a specific range, lipid accumulation products and the visceral adiposity index are associated with the onset of obstructive sleep apnea, suggesting that maintaining ideal lipid accumulation product and visceral adiposity index levels has potential clinical significance in reducing the risk of onset of obstructive sleep apnea.^[24]^ Obesity leads to plasma and hepatic hyperlipidemia and the activation of inflammatory cytokines (TNF-α, IL-6, etc) and transcription factors.^[25]^ These inflammatory factors are associated with sleep disorders. Obesity-induced hypertension also leads to target organ damage.^[26]^

The relationship between sleep disorders and cardiometabolic health may be bidirectional. Sleep disorders such as insomnia and sleep apnea affect the production of a variety of hormones in the body, including insulin, cortisol and leptin, which play key roles in regulating metabolism.^[27]^ For example, sleep deprivation can lead to decreased insulin sensitivity and an increased risk of type 2 diabetes.^[28]^ At the same time, elevated blood pressure after sleep reduction is secondary to sympathetic activation, as evidenced by elevated plasma norepinephrine and heart rate variability indices.^[29]^ The Pennsylvania State University dataset showed that insomniacs who slept < 5 hours had a fivefold greater risk of developing hypertension than did those who slept normally, whereas insomnia with a normal sleep schedule did not portend a significant increase in risk.^[30]^

Emotional and mental health also play important roles in the link between sleep and cardiometabolic health. Sleep deprivation, sleep disruption and their combination increase the risk of future depressive symptoms in older adults.^[31]^ Treating insomnia in depressed patients has a positive effect on mood.^[32]^ Elevated CMI is associated with an increased risk of depression, and addressing dyslipidaemia and improving lipid levels may reduce the risk of depression.^[33]^

In summary, the relationship between cardiometabolic health and sleep quality is complex and multifaceted and involves multiple physiological, metabolic, and psychological factors. Understanding these mechanisms is essential for developing comprehensive interventions, and the use of the CMI to indicate the risk of developing sleep disorders has an important role in this endeavor.

## 5. Limitations

Although we utilized a nationally representative sample, there are several limitations to this study. As a cross-sectional study, we were unable to determine a causal relationship between the CMI and the risk of developing sleep disorders. Sleep disorders were assessed by self-reports, and reporting bias may have occurred. In addition, we could not fully control for all potential confounders, such as mental health status and substance use, which may also influence sleep quality.

## 6. Conclusions

The results of our study revealed a significant association between the CMI and the risk of developing sleep disorders, suggesting that individuals with poorer cardiometabolic health are more likely to experience sleep problems. This finding emphasizes the importance of considering cardiometabolic health when managing sleep disorders. The overall health and quality of life of individuals can be improved through integrated health management strategies. Further comprehensive and detailed prospective studies are needed to validate our results.

## Acknowledgements

We thank our colleagues for their assistance with this study.

## Author contributions

**Data curation:** Junyao Xu, Zhisen Xu.

**Investigation:** Zhisen Xu.

**Methodology:** Xuanfang Qian.

**Writing – original draft:** Zhisen Xu.

**Writing – review & editing:** Junyao Xu.
